# Three New Germacrane-Type Sesquiterpenes with NGF-Potentiating Activity from *Valeriana officinalis* var. *latiofolia*

**DOI:** 10.3390/molecules181114138

**Published:** 2013-11-14

**Authors:** Heng-Wen Chen, Li Chen, Bin Li, Hai-Long Yin, Ying Tian, Qiong Wang, Yan-Hua Xiao, Jun-Xing Dong

**Affiliations:** 1 Department of Pharmaceutical Chemistry, Beijing Institute of Radiation Medicine, Beijing 100850, China; E-Mails: chenhengwen@163.com (H.-W.C.); chenli7274@126.com (L.C.); jkYLiBin@hotmail.com (B.L.); yinhailong1983@126.com (H.-L.Y.); Hq6106@gmail.com (Y.T.); wqwangqiongwq@163.com (Q.W.); yanhuaxiao1982@163.com (Y.-H.X.); 2 Institute of Chemical Defense, Beijing 102205, China

**Keywords:** *Valeriana officinalis* var. *latiofolia*, germacrane-type sesquiterpenes, volvalerenal, volvalerenic acid, PC 12D, NGF-potentiating activity

## Abstract

Three new germacrane-type sesquiterpenoids, volvalerenal F (**1**), volvalerenal G (**2**) and volvalerenic acid D (**3**), along with five known compounds **4**–**8**, were isolated from the CHCl_3_ soluble partition of the ethanol extract of *Valeriana officinalis* var. *latiofolia*. The structures of the new compounds were determined on the basis of spectroscopic evidence, including their 1D- and 2D-NMR spectra, as well as mass spectrometry. The eight germacrane-type sesquiterpenoids showed nerve growth factor (NGF) potentiating activity, which mediates the neurite outgrowth in PC 12D cells. This study intends to reveal the chemical basis of the use of *V. officinalis* var. *latiofolia* as a dietary supplement.

## 1. Introduction

The genus *Valeriana*, from the family *Valerianaceae*, consists of about 250 species widely distributed all over the World. *Valeriana* is a perennial herb native to Europe, Asia and North America [1,2]. Eleven out of 28 *Valeriana* genus species (including one variant) found in China are traditionally used as medicines. Their dried underground parts (roots and rhizomes) exhibit anodyne, antiphlogistic, expectorant and antiasthmatic activities [3,4,5].

Valerians have been used clinically as tranquillizers for the treatment of nervousness, agitation and as a mild sedative to improve sleep [6,7,8,9,10,11,12,13]. Previous studies reported that *Valeriana* contains numerous chemical constituents, including volatile oil, iridoids, flavones, alkaloids, amino acids, and lignans, *etc*. [14,15,16,17,18,19,20]. Valerian (*Valeriana officinalis Linn*.) has been included in the pharmacopoeias in Europe and the United States [21,22], its extracts are sold as dietary supplements and were listed among the top 10 best-selling herbal supplements in the United States in 2002 [23]. Valerian is also widely used as a precious perfume added into food, drink, cosmetics, tobacco, and so on. With significant medicinal and commercial value, it will surely deserve future development.

*V. officinalis* var. *latiofolia*, a variant of *V. officinalis Linn*, is produced mainly in the Guizhou, Sichuan and Yunnan provinces in Southwest China. Particularly in Guizhou, *V. officinalis* var. *latiofolia* is widely cultivated and has become one of the local industry pillars [1,24]. This variant shares some common pharmaceutical actions and chemical constituents with *V. officinalis Linn* [25]. Abundant research has been done on *V. officinalis* var. *latiofolia* so far, and it is assumed that the sesquiterpenoids from the volatile oil and iridoids are the main contributors to its antidepressant and antinervousness activities [24,25]. Pairs of active compounds have been isolated and identified from *Valeriana*, including germacrane-type sesquiterpenoids, volvalerenals A–E, volvalerenic acids A–C, valerianin A–B and heishuixiecaoline A–C [13,16,17,18,19]. However, neither the active component(s) responsible for the therapeutic properties of *Valeriana* nor the related molecular mechanisms are clearly understood, which severely hinders the wider application of *Valeriana* products. Therefore, in this paper the chemical constituents of *V. officinalis* var. *latiofolia* have been systematically investigated, and eight germacrane-type sesquiterpenoids (including three new compounds, volvalerenal F (**1**), volvalerenal G (**2**) and volvalerenic acid D (**3**)) were isolated and identified from chloroform extracts of this herb’s syrup. Additionally, the NGF-potentiating activities of the obtained products were evaluated.

## 2. Results and Discussion

Volvalerenal F (**1**) was isolated as a colorless oil. High-resolution mass spectrometry (HR-ESI-MS) (*m/z* 271.1678 [M+Na]^+^, calcd. for C_16_H_24_O_2_Na, 271.1669) of **1** indicated that its molecular formula is C_16_H_24_O_2,_ and together with the NMR data (Table 1), implied five unsaturated degrees in the molecule. The IR spectrum indicated the presence of carbonyl (1730 cm^−1^) and carbon-carbon double-bond (1627 cm^−1^) absorptions. The ^1^H-NMR spectrum of **1** (Table 1) displayed three olefinic protons at δ_H_ 5.27 (1H, dd, *J* = 5.4, 10.2 Hz, H-1), 5.34 (1H, ddd, *J* = 9.0, 9.0, 3.2 Hz, H-4), and 5.21 (1H, dd, *J* = 9.0, 9.0 Hz, H-5) indicating the presence of two double bonds, methylene proton peaks at δ_H_ 4.42 (1H, d, *J* = 12.0 Hz, H-14a) and δ_H_ 4.21 (1H, d, *J* = 12.0 Hz, H-14b), and three methyls at δ_H_ 0.98 (3H, s, 13-CH_3_), 1.04 (3H, s, 12-CH_3_) and 2.01 (3H, s, 16-CH_3_). Considering the DEPT spectra, the ^13^C-NMR spectrum of **1** (Table 1) suggested the existence of an acetate carbonyl carbon at δ_C_ 171.3 (C-15), four olefinic carbons at δ_C_ 128.8 (C-1), 133.1 (C-10), 128.3 (C-4) and 129.6 (C-5), as well as five methylenes at δ_C_ 28.2 (C-2), 26.8 (C-3), 23.0 (C-8), 35.3 (C-9) and 61.7 (C-14), two methines at δ_C_ 26.4 (C-6) and 31.9 (C-7) and three methyls at δ_C_ 15.6 (C-13), 21.1 (C-16) and 28.9 (C-12).

**Table 1:** ^1^H and ^13^C-NMR Spectroscopic Data of Compounds **1**–**3**.

NO.	1		2		3	
	δ_C_	δ_H_	δ_C_	δ_H_	δ_C_	δ_H_
1	128.8	5.27 *dd* (5.4, 10.2)	128.2	5.22 *dd* (5.4, 11.4)	130.7	5.33 *dd* (5.4, 11.4)
2a	28.2	2.23 *m* H-α	28.3	2.22 *m* (H-α)	29.2	2.38 *m* (H-β)
2b		2.11 *m* H-β		2.15 *m* (H-β)		2.26 *m* (H-α)
3a	26.8	2.14 *m* H-β	24.0	2.71 *dt* (12.0, 4.2, H-α)	27.1	2.71 *dt* (12.6, 4.0, H-α)
3b		2.08 *m* H-α		2.05 *td* (12.6, 4.2, H-β)		2.17 *td* (12.6, 4.0, H-β)
4	128.3	5.34 *ddd* (9.0, 9.0, 3.2)	143.9		132.1	
5	129.6	5.21 *dd* (9.0, 9.0)	158.9	6.48 *d* (9.0)	144.8	6.72 *d* (9.6)
6	26.4	1.14 *t* (9.0, H-α)	31.2	1.52 *t* (13.2, 9.0, H-α)	30.0	1.27 *dd* (9.6, 4.0, H-α)
7	31.9	0.47 *m* (H-α)	38.8	1.02 *m* (H-α)	36.7	0.82 *m* (H-α)
8a	23.0	1.73 *m* (H-β)	24.6	1.86 *m* (H-β)	24.7	1.81 *m* (H-β)
8b		0.76 *m* (H-α)		0.90 *m* (H-α)		0.89 *m* (H-α)
9a	35.3	2.39 *m* (H-β)	35.5	2.60 *m* (H-β)	36.2	2.36 *m* (H-β)
9b		1.87 *t* (12.6, α)		1.88 *m* (H-α)		2.25 *m* (H-α)
10	133.1		139.1		134.3	
11	17.2		22.4		21.5	
12	28.9	1.04 *s*	28.7	1.16 *s*	28.9	1.12 *s*
13	15.6	0.98 *s*	16.0	1.20 *s*	16.1	1.14 *s*
14a	61.7	4.42 *d* (12.0)	196.4	9.20 *s*	172.9	
14b		4.21 *d* (12.0)				
15	171.3		59.1	3.69 *d* (12.0)	62.8	4.32 *d* (12.0)
				3.43 *d* (12.0)		4.15 *d* (12.0)
16	21.1	2.01 *s*			172.0	
17					20.8	1.97 *s*

Compound **1** recorded in CDCl_3**.**_ Compounds **2** and **3** recorded in CD_3_OD. ^1^H-NMR recorded at 600 MHz. ^13^C-NMR recorded at 150 MHz.

**Figure 1:**
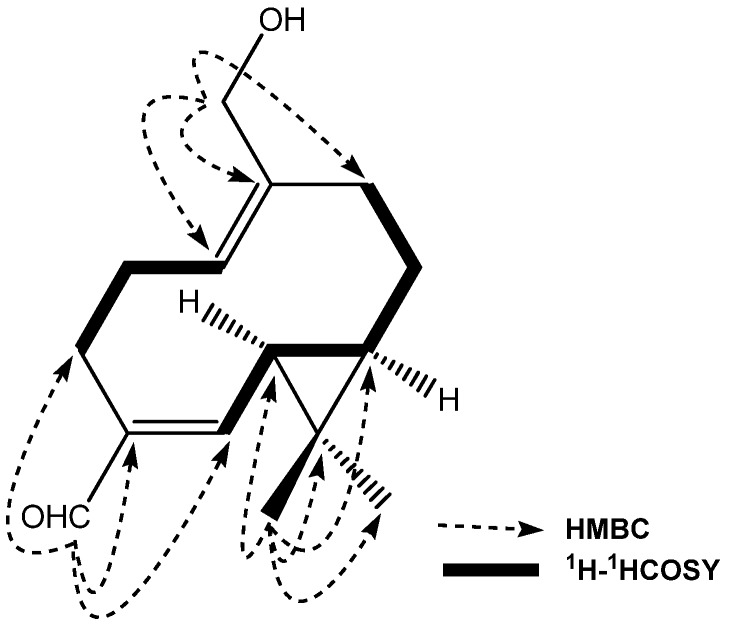
The correlations of structures, ^1^H-^1^H COSY and Key HMBC of **1**.

To confirm the structure of **1**, ^1^H-^1^H COSY and HMBC experiments were conducted (Figure 1), which showed the key correlations such as H-1/H-2, H-2/H-3, H-3 (a,b)/H-4, H-4/H-5, H-5/H-6, H-6/H-7, and H-8/H-9 in its COSY, H-14/C-1, H-14/C-9, H-14/C-10, H-14/C-15, H-3/C-4, H-5/C-4 and H-12/C-6, H-12/C-7, H-12/C-11 and H-12/C-13 in its HMBC. The coupling constant of 9.0 Hz between H-4 and H-5 indicated the *Z-*configuration of the double bond [26,27]. The coupling constant of 9.0 Hz and the NOE correlations between H-6 and H-7 suggested the *syn* and α-oriented of the cyclopropane moiety, and the α-orientation of H-6 and H-7 were assigned by the correlations of H-7/CH_3_-12 and H-6/CH_3_-12 [28]. The correlations of H-2(b)/H-15(a, b) indicated Δ^1,10^ to be *Z* configured. From the above data, the structure of **1** was identified as 14-acetoxy-11,11-dimethylbicyclo[8.1.0]undeca-4*Z* (5),10*Z* (1)-diene, and the compound was named volvalerenal F.

Compound **2** was isolated as a colorless oil. The molecular formula was assigned as C_15_H_22_O_2_ on the basis of HR-ESI-MS from the [M+H]^+^ signal at *m/z* 235.1696 (calcd. for C_15_H_23_O_2_, 235.1693), with five degrees of unsaturation. The IR spectra displayed the presence of carbonyl (1726 cm^−1^), α,β-unsaturated aldehyde (1678 cm^−1^), and carbon-carbon double-bond (1626 cm^−1^) absorptions.

The ^13^C-NMR and DEPT spectra of **2** (Table 1) showed an aldehyde carbon at δ_C_ 196.4 (C-14), four double bond carbons at δ_C_ 128.2 (C-1), 143.9 (C-4), 158.9 (C-5) and 139.1 (C-10), as well as five methylenes (one oxygenated) at δ_C_ 24.0 (C-3), 24.6 (C-8), 28.3 (C-2), 35.5 (C-9) and 59.1 (C-15), two methines at δ_C_ 31.2 (C-6) and 38.8 (C-7), and two methyls at δ_C_ 16.0 (C-13) and 28.7 (C-12). These data suggested that compound **2** was also a germacrane-type sesquiterpenoid. Its ^1^H-NMR spectrum displayed an aldehydic proton obviously at δ_H_ 9.20 (1H, s, H-14), an oxygenated methine proton at δ_H_ 3.69 (1H, d, *J* = 12.0 Hz, H-15a) and 3.43 (1H, d, *J* = 12.0 Hz, H-15b). Above data indicated **2** was structurally similar to heishuixiecaoline B reported in the literature [13].

**Figure 2:**
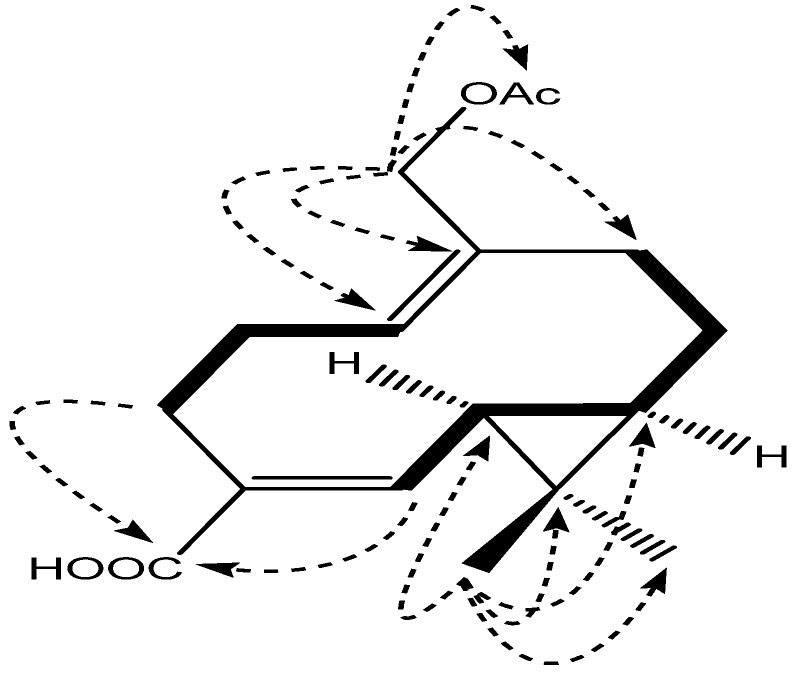
The correlations of structures, ^1^H-^1^H COSY and Key HMBC of **2**.

The proposed structure was further confirmed by HMBC correlations (Figure 2). Key long-range correlations were observed between H-12/C-6, H-12/C-7, H-12/C-11, H-12/C-13, H-15/C-1, H-15/C-10, H-15/C-9, and H-14/C4, H-14/C-5, which suggested the aldehyde group and hydroxyl group were located to C-4 and C-15, respectively. The α-orientation of H-6 and H-7 were assigned as being the same as those of **1** by the correlations of H-6/H-7, H-7/CH_3_-12 and H-6/CH_3_-12 in the NOESY experiment (Figure 4), and the *E*- and *Z*- configurations of Δ^4,5^ and Δ^1,10^ were determined to be the same as in compound **1** by the correlations of H-5/H-14, H-2 (a, b)/H-15 and H-5 with H-1.

On the basis of the above data, the structure of **2** was identified as 4-formyl-10-hydroxymethyl-11, 11-dimethylbicyclogermacren-4*E* (5), 10*Z* (1)-diene, and the product was named volvalerenal G.

Volvalerenic acid D (**3**) was also isolated as a colorless oil. The HR-ESI-MS of **3** indicated that its molecular formula is C_17_H_24_O_2_ (*m/z* 293.1744 [M+H]^+^, calcd. for 293.1747). The IR data was completely similar to that of compound **1**, which suggested that **3** was also a germacrane-type sesquiterpenoid.

**Figure 3:**
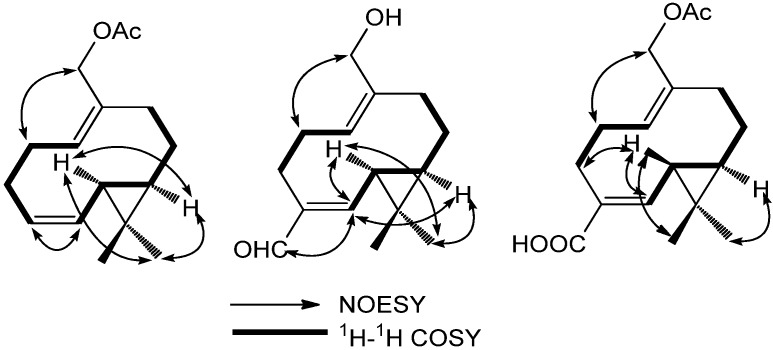
The correlations of structures, ^1^H-^1^H COSY and Key HMBC of **3**.

The NMR spectrum of compound **3** (Table 1) showed the following signals: an acetate carbonyl carbon, four olefinic carbons, five methylenes (one oxygenated), two methines and three methyls, which were quite similar to those of compound **1**. In addition, it is noteworthy that the NMR data displayed an obvious carboxyl carbon signal at δ_C_ 172.0 (C-16). The ^1^H-^1^H COSY spectrum showed key correlations such as H-6/H-7 and H-8/H-9, and key long-range correlations were observed in the HMBC experiments between H-3/C-14 and H-5/C-14 (Figure 3), which suggested the carboxyl group was located to C-4.

**Figure 4:**
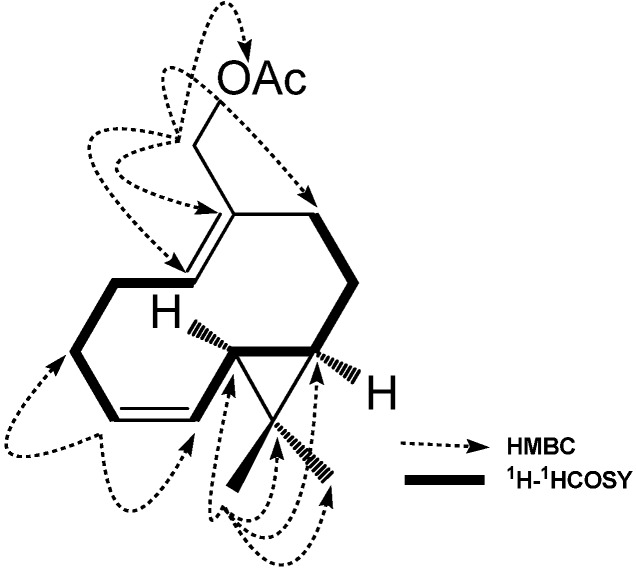
NOE correlations of compounds **1**–**3**.

The NOESY correlations (Figure 4), the coupling constant of 4.0 Hz and the NOE correlations between H-6 and H-7 suggested a *trans* geometry around the cyclopropane ring, and the correlations of H-6/H-3a, H-2 (a, b)/H-15 suggested the *E*- and *Z*- configuration of Δ^4,5^ andΔ^1,10^.

From the above data, the structure of **3** was identified as 15-acetoxy-4-carboxy-11, 11-dimethyl-bicyclogermacren-4*E* (5), 10*Z* (1)-diene, and it compound was named Volvalerenic acid D.

**Figure 5:**
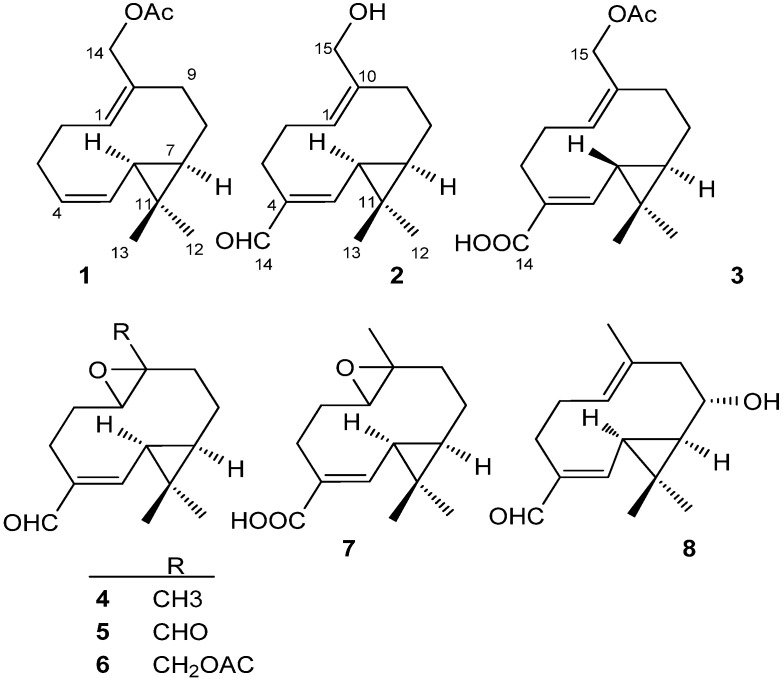
The structures of compounds **1**–**8**.

The five known compounds were identified as madolin A (**4**) and B (**7**) [26], vovalerenal A (**6**) and B (**5**) [18] and heishuixiecaoline B (**8**) [13] by comparing their NMR spectroscopic data with the literature values. The structures of compounds **1**–**8** are shown in Figure 5.

The propensity of compounds **1**–**8** to enhance the activity on nerve growth factor (NGF)-mediated neurite outgrowth in PC 12D cells was assessed as described previously [25]. The neurite-bearing cells accounted for 22.74% and 100% in the control experiments incubated with 2 and 50 ng/mL NGF after 48 h, respectively.

Under 2 ng/mL NGF conditions, all eight tested compounds (at 10, 30, 100 µmol) showed NGF-potentiating activities in various levels. Compound **3** (at 100 µmol) reached 50.15%, in particular (Table 2).

**Table 2:** Effects of compounds **1**–**8** on the proportion of neurite-bearing PC 12D cells in the presence of NGF.

Compound	NGF(ng/mL)	Cell viability (%)		
		10 µmol	30 µmol	100 µmol
**1**	2	24.85 ± 0.98	33.97 ± 1.77 ^b^	43.61 ± 2.11 ^c^
**2**	2	24.42 ± 1.12	35.34 ± 1.48 ^b^	44.30 ± 1.85 ^c^
**3**	2	26.48 ± 0.89 ^a^	37.51 ± 1.66 ^b^	50.15 ± 2.23 ^c^
**4**	2	23.50 ± 1.26	29.33 ± 0.88 ^b^	33.87 ± 1.63 ^c^
**5**	2	24.37 ± 1.01	30.12 ± 1.97 ^b^	40.79 ± 1.17 ^c^
**6**	2	24.74 ± 1.47	34.84 ± 2.35 ^b^	44.15 ± 2.19 ^c^
**7**	2	24.45 ± 1.01	33.96 ± 1.13 ^b^	46.69 ± 2.14 ^c^
**8**	2	24.26 ± 0.73	33.79 ± 1.17 ^b^	49.25 ± 1.25 ^c^
	2	23.08% ± 1.28		
	50	100%		
	0	3.12% ± 0.88		

Results expressed as mean ± SD (n = 6) of eight independent experiments. The 100% value was obtained from 50 ng/mL NGF in the absence of compounds. A statistically significant difference (a, b and c, *p* < 0.01) from the control (2 ng/mL NGF) in the absence of compounds was apparent.

## 3. Experimental

### 3.1. General

Optical rotations were measured with a Perkin-Elmer 343 polarimeter (Perkin-Elmer, Waltham, MA, USA). IR spectra were recorded on the Bio-Rad FTS-65A spectrometer (Bio-Rad, Richmond, VA, USA). UV spectra were recorded using the UV-2501PC spectromter (Shimadzu, Japan). ^1^H and ^13^C-NMR spectra were obtained on a JNM-ECS400 MHz spectrometer (JEOL, Tokyo, Japan) and a Varian UNITY INOVA 600 spectrometer (Varian, Palo Alto, CA, USA), and the chemical shifts were given on δ (ppm) scale with TMS as an internal standard. The HR-ESI-MS were recorded on a 9.4-TQ-FT-MS Apex Qe (Bruker Co., Billerica, MA, USA). Silica gel (60–120 mesh, 200–300 mesh, Qingdao Marine Chemical Group Co., Qingdao, China), and Sephadex LH-20 (Pharmacia, Uppsala, Sweden) were employed for column chromatography. HPLC was carried out using Waters 600E system (Waters, Milford, MA, USA): an analytical column, ODS (5 µm, 4.6 × 250 mm, Hanbon Science & Technology Co., Ltd, Huaian, China), preparative column, a YMC C18 (5 µm, 20.0 × 250 mm, YMC, Kyoto, Japan), detector, Alltech ELSD (evaporative lightscattering detector, Alltech, Los Angeles, CA, USA) 2000ES. Flash chromatography was carried out on Teledyne ISCO Combi Flash *Rf* with Prepacked 80 g silica gel (200–300 mesh) columns (Teledyne Isco, Lincoln, NE, USA). TLC was carried out using silica gel 60 (>230 mesh, Qingdao Marine Chemical Group Co.) and GF254 plates precoated with silica gel 60. Spots on TLC were visually observed under UV light and/or by spraying with anisaldehyde-H_2_SO_4_ reagent followed by heating.

### 3.2. Plant Material

The dry roots of *V. officinalis* var. *latifolia* were collected from the Jiangkou region of Guizhou Province, China, in April 2012. The plant was identified by Prof. Bin Li (Beijing Institute of Radiation Medicine), and a voucher specimen (KYXC-20120313) is deposited in the herbarium of the Beijing Institute of Radiation Medicine, Beijing, China.

### 3.3. Extraction and Isolation

The air-dried roots of *V. officinalis* var. *latifolia* (50 kg) were exhaustively refluxed three times with 60% EtOH (400 L) to give a residue (11 kg) after removal of solvent under reduced pressure. The EtOH extract was suspended in H_2_O and then partitioned successively with CHCl_3_ (3 × 10 L). The CHCl_3_ extract (152.2 g) was subjected to silica gel (200–300 mesh) column chromatography, eluted with petroleum ether-acetone (from 100:1, 75:1, 50:1, 30:1, 25:1, 20:1, 15:1, 10:1, 7:1, 5:1, 3:1, 2:1 and 1:1, v/v) to afford thirteen fractions (A–M). Fraction E (4.711 g) was subjected to flash silica gel chromatography column (80 gram flash column, 60 m L/min) with CHCl_3_/CH_3_OH (from 50:1 to 10:1) to yield eight fractions, E1-E8. Fractions E2-E3 (0.225g) was chromatographed by Sephadex LH-20 (2 × 120 cm, CHCl_3_/CH_3_OH, 1:1) to obtain compound **1** (33 mg). Fractions E4-E7 (0.818 g) was subjected to flash silica gel chromatography (40 gram flash column, petroleum ether-acetone, 5:1, 30 mL/min) and purified by Sephadex LH-20 (2 × 120 cm, CHCl_3_/CH_3_OH, 1:1) to afford compound **6** (86 mg). Compound **4** (168 mg) was isolated from fration E8 by a series of repeated Sephadex LH-20 (2 × 150 cm, CHCl_3_-CH_3_OH, 1:1) column chromatography fractionations. Fraction I (3.678 g) was separated chromatographically on flash silica gel column (80 gram flash column, 60 mL/min) with CHCl_3_/CH_3_OH (40:1 to 2:1), and a total of 50 tubes (15 mL each) were collected. Tubes 19–40 (2.245 g) were chromatographed by flash silica gel chromatography (40 gram flash column, petroleum ether-acetone, 4:1, 30 mL/min) to obtain compound **8** (tubes 10-13, 78 mg). Fraction J (6.366 g) was subjected to a series of purification steps using flash silica-gel column chromatography (80 gram flash column, CHCl_3_/CH_3_OH, 20:1 to 1:1, 60 mL/min) to give ten fractions (J1-J8). Fraction J2-J4 (0.167 g) was chromatographed by Sephadex LH-20 column chromatography (2 × 150 cm, CH_3_OH), and purified by preparative HPLC (CH_3_OH/H_2_O, 75:25, flow rate: 2.0 mL・min^−1^) to afford compounds **2** (68 mg) and **5** (35 mg). Compounds **3** (31 mg) and **7** (78 mg) were obtained from fraction J6-J8 (0.167 g) by preparative HPLC (CH_3_OH-H_2_O, 50:50, flow rate: 2.0 mL・min^−1^).

### 3.4. Compound Characterization

*Volvalerenal F* (**1**): colorless oil. 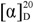 +20.0 (c 0.8, CHCl_3_); UV (CHCl_3_) λ_max_ 237 nm; IR (film) *ν_max_* 3445, 3171, 2960, 2924, 2852, 1730, 1627, 1261, 1095, 1024 cm^−1^; ^1^H-NMR (CHCl_3_, 600 MHz) data, see Table 1; ^13^C-NMR (CHCl_3_, 150 MHz) data, see Table 1; HR-ESI-MS *m/z* 271.1678 [M+Na]^+^ (calcd. for C_16_H_24_O_2_Na, 271.1669).

*Volvalerenal G* (**2**): colorless oil. 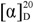 +53.9 (c 0.1, MeOH); UV (CHCl_3_) λ_max_ 264 nm; IR (film) *ν_max_* 3382, 2933, 2864, 1618, 1298, 1190, 1072, 921, 793 cm^−1^; ^1^H-NMR (CHCl_3_, 600 MHz) data, see Table 1; ^13^C-NMR (CHCl_3_, 150 MHz) data, see Table 1; HR-ESI-MS *m/z* 235.1696 [M+H]^+^ (calcd. for C_15_H_23_O_2_, 235.1693).

*Volvalerenic Acid D* (**3**): colorless oil. 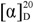 +8.5 (c 0.47, MeOH); UV (CHCl_3_) λ_max_ 240 and 271 nm; IR (film) *ν_max_* 3384, 3245, 2931, 2862, 1238, 1118, 1027, 862, 768 cm^−1^; ^1^H-NMR (CHCl_3_, 600 MHz) data, see Table 1; ^13^C-NMR (CHCl_3_, 150 MHz) data, see Table 1; HR-ESI-MS *m/z* 293.1744 [M+H]^+^ (calcd. for C_17_H_25_O_2_, 293.1747).

### 3.5. Activity Screening *in Vitro*

PC 12D cell line was obtained from Insitute of Biochemistry and Cell Biology, CAS. It was cultured in Dulbecco’s modified Eagles Medium (DMEM, Gibco, New York, NY, USA) with 10% fetal calf serum (Gibco, New York, NY, USA), and 5% equine serum (Gibco), and then the cells were maintained at 37.0 °C in a humidified atmosphere which contained 6% CO_2_ [25]. The test cell line was seeded in 24-well culture paltes (2 × 10^4^ cells/well) coated with poly-L-lysine (Gibco). After 24 h, the medium was changed to test medium containing 1% fetal calf serum, 2% equine serum and varying concentrations of NGF (50 ng/mL for positive control, 2 ng/mL for test samples and significant difference control, Sigma, St. Louis, MO, USA) and test compounds 1–8 (10, 30, 100 µmol). After incubating for 48 h, the cells were fixed with 1% glutaraldehyde (Sigma) in phosphate buffer, and the cells with neurites outgrowth were counted (with at least 100 cells examined/viewing area, three viewing areas/well, six wells/sample). The ratio of the neurite-bearing cells to total cells was determined and expressed as a percentage.

## 4. Conclusions

Three new germacrane-type sesquiterpenoids, volvalerenal F (**1**), volvalerenal G (**2**) and volvalerenic acid D (**3**), along with five known compounds **4**–**8**, were isolated from the CHCl_3_ soluble partition of the ethanol extract of *Valeriana officinalis* var. *latiofolia*. NGF plays a key role in the functions of the central and peripheral nervous system [29] and all the sesquiterpenoids obtained displayed certain NGF-potentiating activities. From the current investigation it can be predicted that sesquiterpenoids will be promising candidates for dietary supplements and medicines, although further studies are needed to determine the pharmacological activities and the mechanism of these eight compounds in animals.
